# Complete Genome Sequence of an Avian Native NDM-1-Producing Salmonella enterica subsp. enterica Serovar Corvallis Strain

**DOI:** 10.1128/genomeA.00593-18

**Published:** 2018-06-28

**Authors:** Sead Hadziabdic, Maria Borowiak, Angelina Bloch, Burkhard Malorny, Istvan Szabo, Beatriz Guerra, Annemarie Kaesbohrer, Jennie Fischer

**Affiliations:** a Department for Biological Safety, German Federal Institute for Risk Assessment (BfR), Berlin, Germany; b European Food Safety Authority (EFSA), Parma, Italy

## Abstract

Carbapenems are an important class of β-lactams and one of the last options for treating severe human infections. We present here the complete genome sequence of avian native carbapenemase-producing Salmonella enterica subsp. enterica serovar Corvallis strain 12-01738, harboring a *bla*_NDM-1_-carrying IncA/C_2_ plasmid, isolated in 2012 from a wild bird (Milvus migrans) in Germany.

## Abstract (precis)

Carbapenems are an important class of β-lactams and one of the last options for treating severe human infections. We present here the complete genome sequence of avian native carbapenemase-producing Salmonella enterica subsp.

## GENOME ANNOUNCEMENT

Antimicrobial resistance in bacterial populations among food-producing animals presents an important concern for public health (1). Therefore, carbapenems are classified as “critically important” antimicrobials and one of the last options for treating severe human infections caused by multidrug-resistant bacteria (2). In recent years, carbapenemase-producing *Enterobacteriaceae* from different nonhuman matrices were sporadically detected in Germany (3–5). Through routine diagnostics in 2012, the German National Reference Laboratory for Salmonella received an avian native NDM-1-producing Salmonella enterica subsp. enterica serovar Corvallis strain (12-01738) harboring the *bla*_NDM-1_ gene on an ∼180-kb IncA/C_2_ plasmid (6, 7).

In recent *in vivo* infection studies, we demonstrated the persistence of this strain in broiler chickens and dissemination of its IncA/C_2_ *bla*_NDM-1_-carrying plasmid to different *Enterobacteriaceae*, both without antibiotic pressure (8). Such observations are noteworthy due to a possible scenario of *bla*_NDM-1_ introduction into commercial broiler production and downstream in the production chain, posing a subsequent risk for human exposure. In order to obtain the full-genome sequence, this S. Corvallis strain (12-01738) was submitted to PacBio RS II long-read sequencing.

DNA extraction using the PureLink genomic DNA minikit (Invitrogen, Carlsbad, CA, USA) was followed by PacBio RS II system-based genome sequencing (GATC Biotech AG, Constance, Germany). *De novo* genome assembly was performed using the SMRT Analysis software (version 2.3.0; Pacific Biosciences, USA).

Through additional whole-genome sequencing analysis by Illumina MiSeq technology, we demonstrated the presence of the bacterial chromosome (4,887,378 bp) and, as confirmed by S1-PFGE (S1–pulsed-field gel electrophoresis) plasmid profiling, the presence of three plasmids in this isolate (average coverage, 153.38-fold per consensus base).

Plasmid analysis using the tools available at the Center for Genomic Epidemiology (CGE; http://www.genomicepidemiology.org/) revealed the presence of an IncA/C_2_ NDM-1-encoding plasmid (pSE12-01738-2; 177,190 bp), two additional plasmids of incompatibility group IncHI2 (sequence type 1 [ST-1]; pSE12-01738-1; 284,485 bp), and a ColE-like (ColRNAI) plasmid (pSE12-01738-3; 10,047 bp). The pSE12-01738-2 plasmid is a derivative of the *bla*_NDM-1_-carrying IncA/C_2_ multiresistance plasmid pRH-1238 (GenBank accession number KR091911), previously described by Villa et al. (7) but lacking a genetic element including genes for chromate and macrolide resistance as well as a class I integron carrying *dfrA7-aadA5-sul1* resistance gene cassettes. This, along with previously observed structural deletions in pRH-1238 after an *in vivo* passage, indicates certain evolutionary plasticity, still enabling maintenance of the *bla*_NDM-1_ gene (8).

CGE-based resistome analysis revealed that the IncHI2 plasmid pSE12-01738-1 harbors two resistance genes [*aac(6')Ib-cr* and *aacA4*], also located on the IncA/C_2_ plasmid pSE12-01738-2, which itself harbors 16 resistance genes. The ColE-like (ColRNAI) plasmid pSE12-01738-3 harbors only a *qnrS1* gene. The genome was annotated using the automated Prokaryotic Genome Annotation Pipeline (https://www.ncbi.nlm.nih.gov/genome/annotation_prok/), where the presence of 5,177 coding sequences, 235 pseudogenes, and 123 RNA genes (22 rRNAs, 84 tRNAs, and 17 noncoding RNAs) was observed on the bacterial chromosome.

### Accession number(s).

These sequences were deposited in GenBank under the accession numbers CP027677 (chromosome), CP027678 (pSE12-01738-1), CP027679 (pSE12-01738-2), and CP027680 (pSE12-01738-3).
